# A Comparative Study of Various Pretreatment Approaches for Bio-Ethanol Production from Willow Sawdust, Using Co-Cultures and Mono-Cultures of Different Yeast Strains

**DOI:** 10.3390/molecules27041344

**Published:** 2022-02-16

**Authors:** Imen Ben Atitallah, Georgia Antonopoulou, Ioanna Ntaikou, Amaia Soto Beobide, Vassilios Dracopoulos, Tahar Mechichi, Gerasimos Lyberatos

**Affiliations:** 1 Laboratory of Biochemistry and Enzyme Engineering of Lipases, National School of Engineers of Sfax, University of Sfax, BP 1173, Sfax 3038, Tunisia; benatitallahimen@gmail.com (I.B.A.); tahar.mechichi@enis.rnu.tn (T.M.); 2 Institute of Chemical Engineering Sciences (FORTH/ICE-HT), Stadiou, Platani, GR 26504 Patras, Greece; ntaikou@iceht.forth.gr (I.N.); asoto@iceht.forth.gr (A.S.B.); indy@iceht.forth.gr (V.D.); lyberatos@chemeng.ntua.gr (G.L.); 3 School of Chemical Engineering, National Technical University of Athens, GR 15780 Athens, Greece

**Keywords:** willow sawdust, alkaline, peroxide, pretreatment, ethanol, *Saccharomyces cerevisiae*, *Pichia stipites*, *Pachysolen tannophilus*, *Wickerhamomyces anomalus X19*, hydrolysis, whole slurry

## Abstract

The effect of different pretreatment approaches based on alkali (NaOH)/hydrogen peroxide (H_2_O_2_) on willow sawdust (WS) biomass, in terms of delignification efficiency, structural changes of lignocellulose and subsequent fermentation toward ethanol, was investigated. Bioethanol production was carried out using the conventional yeast *Saccharomyces cerevisiae,* as well as three non-conventional yeasts strains, i.e., *Pichia stipitis*, *Pachysolen tannophilus, Wickerhamomyces anomalus X19*, separately and in co-cultures. The experimental results showed that a two-stage pretreatment approach (NaOH (0.5% *w*/*v*) for 24 h and H_2_O_2_ (0.5% *v*/*v*) for 24 h) led to higher delignification (38.3 ± 0.1%) and saccharification efficiency (31.7 ± 0.3%) and higher ethanol concentration and yield. Monocultures of *S. cerevisiae* or *W. anomalus X19* and co-cultures with *P. stipitis* exhibited ethanol yields in the range of 11.67 ± 0.21 to 13.81 ± 0.20 g/100 g total solids (TS). When WS was subjected to H_2_O_2_ (0.5% *v*/*v*) alone for 24 h, the lowest ethanol yields were observed for all yeast strains, due to the minor impact of this treatment on the main chemical and structural WS characteristics. In order to decide which is the best pretreatment approach, a detailed techno-economical assessment is needed, which will take into account the ethanol yields and the minimum processing cost.

## 1. Introduction

Due to the depletion of fossil fuel resources, fluctuations in the price of crude oil, and an increased concern for environmental pollution, the development of clean, renewable and sustainable energy is crucial [1,2]. In this context, recent progress in the energy sector has focused on the technological conversion of lignocellulosic biomass toward biofuels, including bioethanol [3]. The valorization of agricultural wastes [4,5,6], forestry residues [1,7] and weedy biomass [8] toward second generation bioethanol production [9] has attracted significant attention during the previous decades, aiming to provide a sustainable solution for the reduction of energy dependence on fossil-based fuels, without being antagonistic to food/feed resources and the environmental problems associated with the reliance on them [10].

Willow (*Salix* sp.) sawdust (WS) is an abundant feedstock of practically zero cost that could serve as a promising raw material for bioethanol production due to its high hollocellulosic content. However, the recalcitrant nature of WS might hamper polysaccharide accessibility for enzymatic hydrolysis to fermentable sugars for subsequent use in fermentation processes. The latter, however, might be greatly facilitated by the selection of a proper pretreatment method [11,12]. Pretreatment represents a crucial step for the efficient fractionation of biomass components and enzymatic hydrolysis processes, being responsible for the removal of the lignin seal of hollocellulose, as well as the reduction of cellulose crystallinity [13]. Removal of lignin or hemicellulose during pretreatment has been shown to greatly improve the performance of cellulolytic enzymes. The negative effect of lignin during enzymatic hydrolysis has been reported in many studies, since lignin is the most recalcitrant component in woody biomass such as poplar [14,15] and willow [16]. Therefore, an effective lignin extraction or disruption could significantly affect the structural matrix and crystallinity of the cellulose and ultimately enhance biomass saccharification during enzymatic hydrolysis. However, the main drawback of most chemical pretreatment methods is the generation of a wide range of toxic byproducts, i.e., phenolic compounds, furanic aldehydes (furfural, 5-hydroxy methyl furfural (HMF)) and weak acids, which might be inhibitory to enzymes and microorganisms [17,18]. Therefore, the minimization of the formation of inhibitory compounds during pretreatment and/or the selection of resistant microbial strains are critical for an efficient fermentation, being thus important for the economic viability of the overall process.

Alkaline pretreatment, through the use of NaOH, has been found to be efficient for the partial lignin removal and hemicellulose solubilization to its oligomers, while cellulose is affected to a smaller degree [12,19]. It is commonly carried out at low temperatures (~80 °C), without releasing furanic compounds in the pretreatment slurry. However, phenolic compounds could be produced due to the lignin degradation that is carried out at high pH values [19]. Conversely, the use of H_2_O_2_ as an oxidative agent, producing free radicals (HO· and HOO·) and molecular oxygen upon decomposition [20] is generally performed at mild operating conditions, providing a high degree of enzymatic hydrolysis efficiency and enhancement of cellulose accessibility, thus leading to high glucose yields [21].

Recently, research has shown that the combined use of sodium hydroxide (NaOH) with hydrogen peroxide (H_2_O_2_) as a pretreatment can be quite effective for a wide range of lignocellulosic biomass types, significantly improving the delignification step and enhancing the enzymatic hydrolysis of hollocellulose [22,23,24]. Furthermore, this type of pretreatment can be effective at relatively low temperatures, requiring only low energy consumption, whereas the generation of inhibitory products is considerably lower compared to other chemical pretreatment methodologies, based on the use of acids and high temperatures [21,25,26].

In general, the lignin removal efficiency achieved by the combination of NaOH with H_2_O_2_ strongly depends on the pH of the treatment, which promotes reactive oxygen radical formation, causing a disruption of the complex matrix of lignocellulose, thereby exposing cellulose and enhancing its dissolution capability [27,28,29]. It has been tested on different lignocellulosic feedstocks, such as corn stover [30], rapeseed straw [31] and rice hulls [32].

A typical biorefinery processing of lignocellulosic biomass involves a pretreatment step prior to subsequent enzymatic hydrolysis, during which biological conversion of carbohydrates into monomeric sugars takes place by using specific enzymes and fermentation of the released sugar solution for the production of biofuels. The latter processes of enzymatic hydrolysis and fermentation could be accomplished either separately (separate hydrolysis and fermentation (SHF)) or simultaneously (simultaneous saccharification and fermentation (SSF)), with certain advantages for both processes [33].

Generally, although *Saccharomyces cerevisiae* remains the world’s most exploited yeast for alcoholic fermentation, its wild strains are incapable of metabolizing the pentose sugars (i.e., xylose and arabinose) that are released during hydrolysis of hemicelluloses [6]. Such sugars can also be fermented to ethanol by other yeast genera, namely C5 strains, with *Pichia* [34], *Candida* [35] and *Pachysolen* [36] being proposed as the most promising for bioethanol production from various lignocellulosic feedstocks, either in mono-culture, or in co-culture with conventional strains. Co-culturing seems to have advantages over mono-cultures [34]. This can be attributed to possible synergistic actions of different strains toward more efficient substrate utilization and bioconversion [37]; the co-culture of *P. stipitis* with *S. cerevisiae* is reported to be as the most efficient since the two species have the same culturing requirements for optimal fermentation [38].

The aim of the present study was to assess the effect of the combination of NaOH with H_2_O_2_ (alkali-hydrogen peroxide)-based pretreatment schemes on WS biomass, in terms of the imposed structural changes of lignocellulose, delignification efficiency and subsequent fermentation to ethanol in SSF mode. In this context, the whole slurries of pretreated WS, obtained after sodium hydroxide and/or hydrogen peroxide pretreatment, alone or in combination, were assessed in a comparative way, for bioethanol production via the conventional yeast *S. cerevisiae* CECT 1332, as well as three non-conventional yeast strains, i.e., *Pichia stipitis* CECT 1922, *Pachysolen tannophilus* CECT 1426 and *Wickerhamomyces anomalus X19 (Pichia anomala* X19), separately and in co-culture. To the best of our knowledge, the proposed pretreatment methodology was applied for the first time to enhance bioethanol production from willow biomass, using the selected yeast strains. Thus, this is the first comparative study assessing the impact of five different pretreatment approaches based on NaOH/H_2_O_2_ in lignocellulosic residue, while simultaneously assessing the impact of using mono- or co-cultures of ethanol producing strains, in its fermentation to bioethanol.

## 2. Results and Discussion

### 2.1. Characterization of WS before and after Pretreatment

#### 2.1.1. Chemical Characteristics

The composition of the raw WS used in the present study is presented in Table 1. As can be observed, WS was mainly composed of cellulose at 33.4 ± 1.1% (g/g TS), while hemicellulose was at 21.5 ± 0.9% (g/g TS) and lignin at 29.1 ± 0.6% (g/g TS). The biomass used had similar chemical composition with the respective previous studies [16,39,40] in which WS was used as substrate for anaerobic digestion. *Salix* composition may vary depending on the varieties, the region of growth and cultivation conditions. In recent years, different fast-growing *Salix* varieties have been developed in northern Europe, ensuring frost tolerance, pest resistance and high biomass productivity [41]. In the study by Sassner et al. [41], wood chips of a *Salix* sp. hybrid were used with 41.4% glucan, 15% xylan and 24.2% lignin (on a dry basis). A higher lignin content (37.4%) was determined in young willow stems containing bark, and it was used for phytoremediation of trace elements [42].

**Figure 1:**
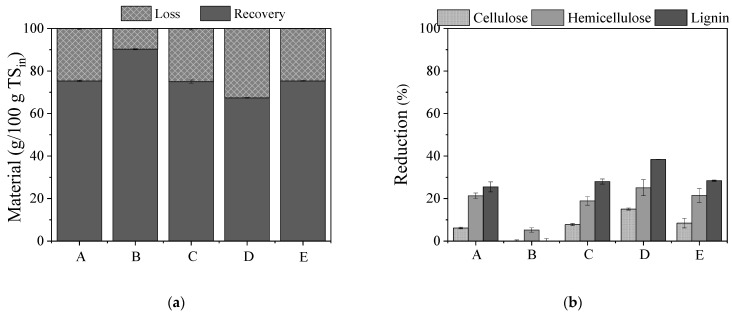
(**a**) Material loss and recovery expressed as g/100g TS_in_ (in: initial biomass); (**b**) reduction of lignocellulosic content during different pretreatment approaches. (A) NaOH (0.5% *w*/*v*), (B) H_2_O_2_ (0.5% *v*/*v*), (C) mixture of NaOH (0.5% *w*/*v*) and H_2_O_2_ (0.5% *v*/*v*) at ratio 1:1, (D) initially NaOH (0.5% *w*/*v*) followed by H_2_O_2_ (0.5% *v*/*v*), and (E) initially H_2_O_2_ (0.5% *v*/*v*) followed by NaOH (0.5% *w*/*v*).

During pretreatment, five different handlings were tested, i.e., one step pretreatment with: (A) NaOH (0.5% *w*/*v*) for 24 h at 80 °C, (B) H_2_O_2_ (0.5% *v*/*v*) for 24 h or (C) mixture of NaOH (0.5% *w*/*v*) and H_2_O_2_ (0.5% *v*/*v*) at ratio 1:1 for 24 h at 80 °C, and two-step processes (D) initially to NaOH (0.5% *w*/*v*) for 24 h at 80 °C, followed by H_2_O_2_ (0.5% *v*/*v*) for 24 h at 80 °C or (E) to H_2_O_2_ (0.5% *v*/*v*) for 24 h at 80 °C first, followed by NaOH (0.5% *w*/*v*) for 24 h at 80 °C. In Figure 1, the material recovery (Equation (1)) and loss (Equation (2)) (Figure 1a) as well as the reduction (%) of the lignocellulosic components (Equation (3)) (Figure 1b) due to pretreatment are presented. As shown, lignin, cellulose and hemicellulose (calculated from Equation (4)) decreased after pretreatment, to a different extent, with the lignin reduction being higher, especially in the case of NaOH (A) or combined NaOH/H_2_O_2_ pretreatment, applied either in a single- (C) or two-step process (D and E). The fact that alkaline pretreatment mainly influences the lignin contained in different lignocellulosic biomasses has extensively been reported [12,43]. The sole application of H_2_O_2_ did not significantly influence the lignin, cellulose and hemicellulose content (*p* > 0.05 for each component before and after pretreatment). This was also confirmed by the high material recovery that was observed, which amounted to 90.2 ± 0.3%. However, its combination with an acidic or alkaline agent could enhance the delignification or depolymerization (of the holocellulose) efficiency, since its effect on the fractionation of the lignocellulosic structure is strongly related to the pH during pretreatment. Based on the literature, addition of an alkali such as NaOH and adjustment of the pH to 11.5 promotes delignification efficiency through lignin side-chain cleavage, breaking its seal to low-molecular compounds [44]. This pretreatment also promotes hemicellulose solubilization while preserving the cellulose fraction [21]. Apart from alkaline addition, combination of H_2_O_2_ with an acid, such as phosphoric or acetic acid, has also been proposed as an efficient pretreatment method for various feedstocks, leading to removal of hemicellulose and lignin as well as disruption of the crystalline cellulose structure [45].

In the present study, hemicellulose was reduced to 21.3%, 18.9%, 25.1% and 21.4% when WS was subjected to pretreatment approaches A (NaOH), C (mixture of NaOH and H_2_O_2_), D (initially NaOH followed by H_2_O_2_) and E (initially H_2_O_2_ followed by NaOH), respectively. Lignin removal was highest (38.3%) for pretreatment approach D, while approaches A, C and E led to lower lignin reduction (25.5%, 27.9% and 28.4%, respectively). The two-sample *t*-test revealed that there were statistical differences between lignin/cellulose/hemicellulose before and after A, C, D and E pretreatments. In addition, by comparing the pretreatments A, C, D and E, the *p* values in the ANOVA results for the lignin, hemicellulose and cellulose content were all <0.05, implying that there were significant differences in the effects of the different pretreatments on lignocellulose fractionation. The same trends were observed for the material recoveries, which were 75.3%, 75.0% and 75.3% for pretreatments A, C and E, respectively, while a lower recovery of 67.4% was observed for pretreatment approach D, confirming the higher solubilization of lignin and hemicellulose that was measured. 

**Figure 2:**
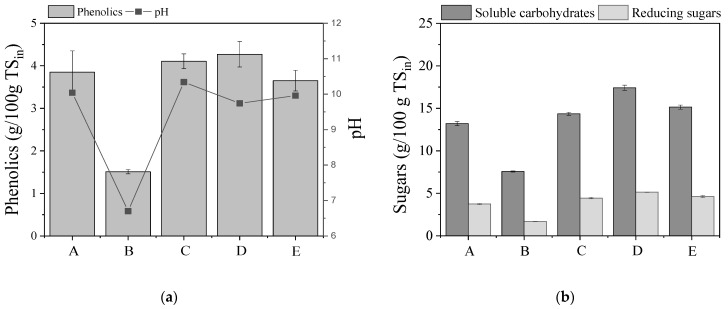
(**a**) Phenolic compound concentration expressed as g/100g TS_in_ (in: initial biomass) (left axis) and pH (right axis). (**b**) Soluble carbohydrates and reducing sugars in the slurries from the different pretreatment approaches. (A) NaOH (0.5% *w*/*v*), (B) H_2_O_2_ (0.5% *v*/*v*), (C) mixture of NaOH (0.5% *w*/*v*) and H_2_O_2_ (0.5% *v*/*v*) at ratio 1:1, (D) initially NaOH (0.5% *w*/*v*) followed by H_2_O_2_ (0.5% *v*/*v*), and (E) initially H_2_O_2_ (0.5% *v*/*v*) followed by NaOH (0.5% *w*/*v*).

The advantage of NaOH/H_2_O_2_ pretreatment compared to the other chemical pretreatments is the absence of furanic toxic compounds, such as furfural and HMF [31]. However, the pH of the process determines the severity of the pretreatment, also correlated with the generation of inhibitors, such as phenolics, which are released during delignification [19]. The phenolic concentration and the pH in the slurries coming from the different pretreatments of the current study are shown in Figure 2a.

As shown in the figures, the concentration of the phenolic compounds, approximately in all cases, was in the range from 3.6 ± 0.2 to 4.3 ± 0.2 g/100g TS. A statistical difference was observed between pretreatments A and D (*p* = 0.00069) and D and E (*p* = 0.024). Conversely, the phenolic compound concentration detected in the slurry obtained from pretreatment B was statistically different compared to the others (*p* < 0.05 in all cases), with a considerably lower value, i.e., 1.5 ± 0.1 g/100g TS. These results are in agreement with the measured values for lignocellulosics reduction and material recovery (Figure 1). As shown from the pH values presented in Figure 2a, all the pretreatment approaches, apart from B, were carried out in alkaline conditions (pH range, 9.7–10.3). The pretreatment approach B was performed in slightly acidic conditions (pH = 6.7) and this could possibly justify its low impact on the lignocellulosic fractionation. As previously mentioned, the combination of H_2_O_2_ with alkali at a pH of approximately 11 leads to high delignification efficiency and solubilization of the holocellulose [44] contained in lignocellulosic feedstock.

Sugar release (total soluble carbohydrates and reducing sugars) due to solubilization of hollocellulose is shown in Figure 2b. The saccharification degree during the pretreatments was determined by the estimation of the fraction of soluble carbohydrates as reducing sugars (% reducing/soluble) (Equation (5)), while the saccharification efficiency (Equation (6)) was the ratio of soluble sugars to the initial holocellulose (g/g). The values of saccharification degree and efficiency are presented in Table 2. Soluble carbohydrate concentration, as well as saccharification efficiency, were higher in the case of the two-stage pretreatment D, which also led to lower material recovery, higher concentration of phenolic compounds and higher reduction of the lignocellulosic fraction. Slightly lower saccharification efficiency values were estimated for pretreatment approaches A, C and D, as seen in Table 2 and Figure 2b, and the lowest saccharification efficiency was observed for the case of pretreatment with H_2_O_2_ (B) (13.8 ± 0.1% g/g). ANOVA results showed that saccharification efficiency of pretreatment approaches A, C, D and E were statistically different.

#### 2.1.2. Structural Characteristics

**Figure 3:**
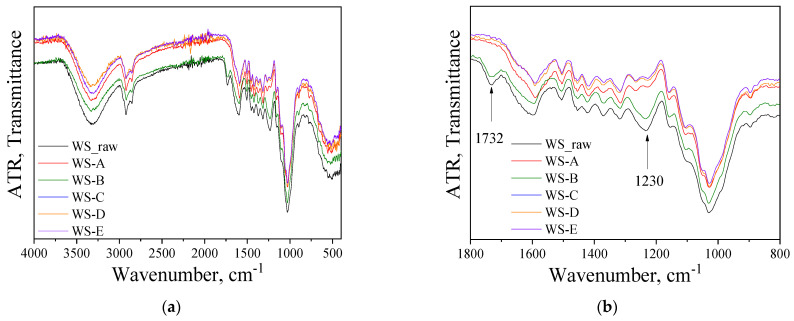
(**a**) FTIR-ATR spectra in the whole range and (**b**) in the fingerprint region between 1800 and 800 cm^−1^ for raw and WS samples after pretreatment approaches with (A) NaOH (0.5% *w*/*v*), (B) H_2_O_2_ (0.5% *v*/*v*), (C) mixture of NaOH (0.5% *w*/*v*) and H_2_O_2_ (0.5% *v*/*v*) at ratio 1:1, (D) initially NaOH (0.5% *w*/*v*) followed by H_2_O_2_ (0.5% *v*/*v*), and (E) initially H_2_O_2_ (0.5% *v*/*v*) followed by NaOH (0.5% *w*/*v*).

FTIR analysis was performed in order to qualitatively examine the structural and chemical changes of lignocellulosics in WS, induced by the various pretreatment approaches applied in the present study. Figure 3a shows the IR spectra of raw and pretreated WS in the whole spectral range, detecting the main functional groups of the components that constitute the lignocellulosic material, while the fingerprint region between 1800 and 800 cm^−1^ is shown in Figure 3b.

The strong absorption band seen in Figure 3a at around 3400 cm^−1^ is assigned to O–H stretching vibrations due to intramolecular hydrogen bonding in phenolic groups and O–H stretching of alcohols, phenols, acids and weakly bounded absorbed water, while the one at around 2900 cm^−1^ is attributable to the C–H groups. Furthermore, there are also many well-defined peaks in the fingerprint region between 1800 and 800 cm^−1^ (Figure 3b). The most representative bands studied within this spectral range are summarized in Table 3.

Due to pretreatment of willow, the absorption peaks of several bonds were reduced, compared to the raw material, indicating chemical changes in the structure of treated samples. All spectra were normalized using the intensity of the reference band at 1024 cm^−1^, assigned to invariant CO stretching. Even if the chosen band did not remain completely constant in all spectra, it represents one of the less variable bands during the treatment [46,47].

With more detailed scrutiny, differences due to pretreatments can be detected in the IR spectra. A decrease in the peak at around 1230 cm^−1^ is observable in almost all the spectra of pretreated samples, except for that of pretreatment B (WS treated with H_2_O_2_), which presents the lowest peak intensity reduction. This IR peak is correlated to the syringyl ring and C–O stretch in lignin [48]. Besides, there is another peak changing its intensity, depending on the pretreatment, and this is the band centered at 1732 cm^−1^, attributed to unconjugated carbonyl stretching in hemicellulose [49]. These two bands (1230 and 1732 cm^−1^) almost disappeared for all pretreatments applied, except for the one performed with H_2_O_2_ alone. According to the analysis, reduction of the bands at 1732 and 1230 cm^−1^ indicates that the quantities of lignin and hemicellulose were clearly reduced during the pretreatments A, C, D and E, confirming the measurements of lignocellulosics as presented in Figure 1. 

**Figure 4:**
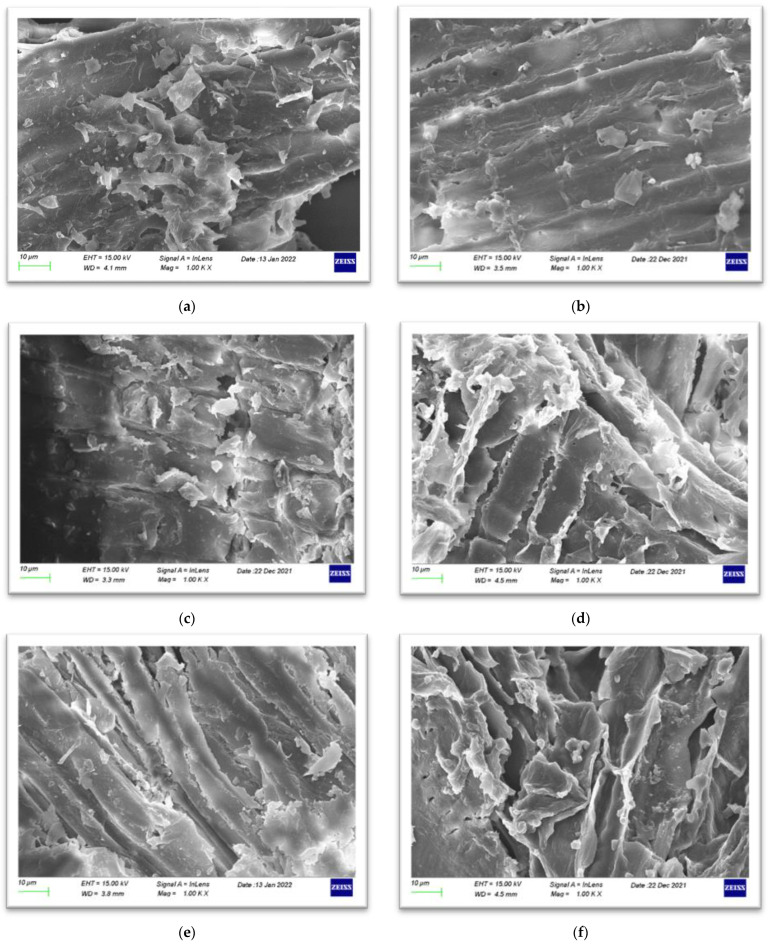
SEM images of (**a**) raw WS and WS after pretreatment with (**b**) (A) NaOH (0.5% *w*/*v*), (**c**) (B) H_2_O_2_ (0.5% *v*/*v*), (**d**) (C) mixture of NaOH (0.5% *w*/*v*) and H_2_O_2_ (0.5% *v*/*v*) at ratio 1:1, (**e**) (D) initially NaOH (0.5% *w*/*v*) followed by H_2_O_2_ (0.5% *v*/*v*), and (**f**) (E) initially H_2_O_2_ (0.5% *v*/*v*) followed by NaOH (0.5% *w*/*v*).

In Figure 4, SEM images of raw and pretreated WS samples are presented. As shown, there is a different surface morphology after application of each pretreatment, revealing the disruption of the lignocellulose, as also confirmed by FTIR-ATR spectra and lignocellulosic fractionation measurements. Raw WS presented a partially ordered structure on its surface, containing repeated slots (Figure 4a), which did not change significantly with the application of sole H_2_O_2_ (Figure 4c). However, the application of NaOH treatment or its combination with H_2_O_2_ (treatments A, C, D and F) caused a distorted view on the outer surface of the WS, compared to the raw samples. Disordered fibrils, tiny holes, larger slots and cavities, as well as parts with a smoother outer layer are some of the pretreated samples’ characteristics (Figure 4b,d–f), caused by the removal of non-cellulosic polymers of WS. Similar observations were reported in other studies, in which NaOH/H_2_O_2_ pretreatment was performed in corn stover [50], rice straw [51] and wheat straw [52]. The modified structure of the pretreated samples promotes the accessibility of the enzymes to the inner cellulose in the following biochemical steps (SSF mode), thus enhancing saccharification efficiency and enzymatic hydrolysis. Finally, the observation of the smoothing of the outer layer of the fiber surface, which is partially evident in all pretreated samples, except for the sample of pretreatment B, is a common characteristic of alkali-pretreated samples, and it is in good agreement with the literature [8,43].

### 2.2. Ethanol Production Experiments 

**Figure 5:**
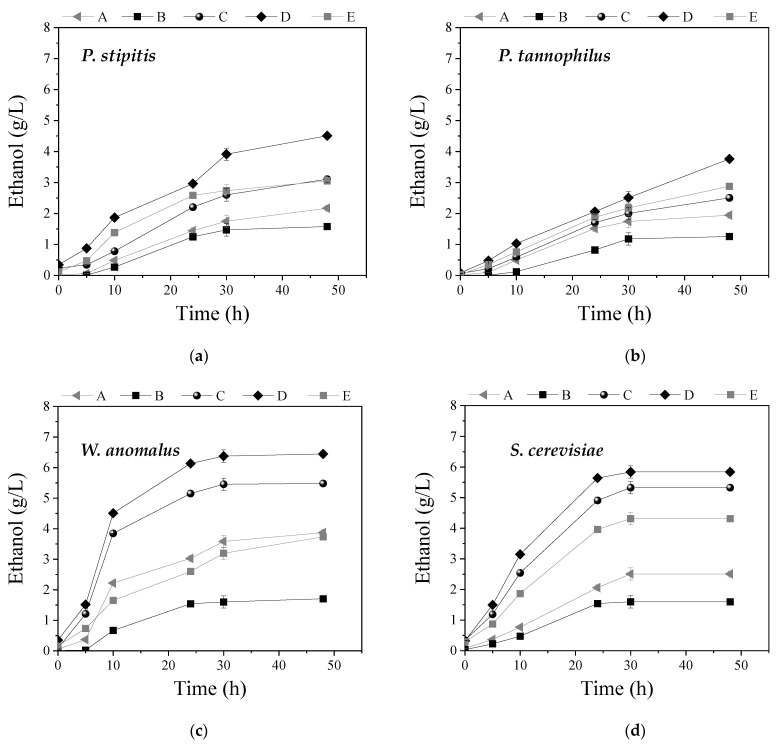
Ethanol concentration during batch mono-culture of (**a**) *Pichia stipitis*, (**b**) *Pachysolen tannophilus*, (**c**) *Wickerhamomyces anomalus* X19, and (**d**) *Saccharomyces cerevisiae* for the fermentation of the whole slurry after pretreatment with (A) NaOH (0.5% *w*/*v*), (B) H_2_O_2_ (0.5% *v*/*v*), (C) mixture of NaOH (0.5% *w*/*v*) and H_2_O_2_ (0.5% *v*/*v*) at ratio 1:1, (D) initially NaOH (0.5% *w*/*v*) followed by H_2_O_2_ (0.5% *v*/*v*), and (E) initially H_2_O_2_ (0.5% *v*/*v*) followed by NaOH (0.5% *w*/*v*).

**Figure 6:**
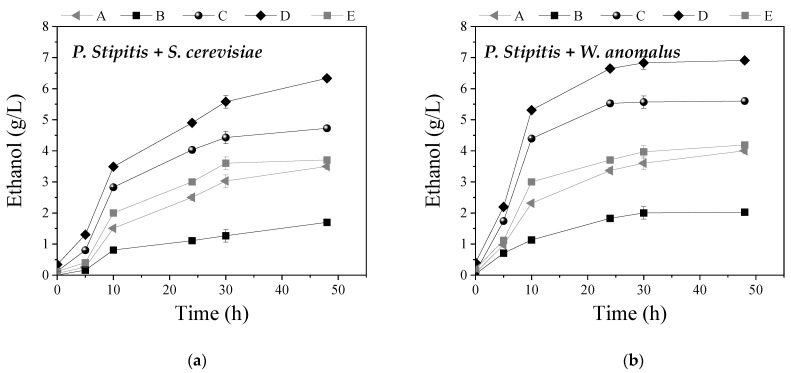
Ethanol concentration during batch co-culture of (**a**) *Pichia stipitis* and *Saccharomyces cerevisiae*, (**b**) *Pichia stipitis* and *Wickerhamomyces anomalus* X19 for the fermentation of the whole slurry after pretreatment with (A) NaOH (0.5% *w*/*v*), (B) H_2_O_2_ (0.5% *v*/*v*), (C) mixture of NaOH (0.5% *w*/*v*) and H_2_O_2_ (0.5% *v*/*v*) at ratio 1:1, (D) initially NaOH (0.5% *w*/*v*) followed by H_2_O_2_ (0.5% *v*/*v*), and (E) initially H_2_O_2_ (0.5% *v*/*v*) followed by NaOH (0.5% *w*/*v*).

In the present study, the whole slurries obtained after WS pretreatment approaches A to E were used for ethanol production via SSF, through the use of a commercial cellulase blend at 30 FPU/g TS and mono-cultures or co-cultures of ethanol-producing yeast strains. Specifically, mono-cultures of the C6 yeast strains *W. anomalus* X19 and *S. cerevisiae* or the well-known C5 yeast strains of *P. stipitis* and *P. tannophilus*, as well as the co-cultures of *P. stipitis* with either *S. cerevisiae* or *W. anomalus* X19, were tested for ethanol production for 48 h of fermentation. The use of *P. stipitis* or *P. tannophilus* was based on the ability of those strains to ferment both C5 and C6 sugars, since the hydrolyzed slurries obtained after pretreatment may contain xylose, coming from hemicellulose solubilization, as also reported previously [8]. The selection of the non-conventional strain *W. anomalus* was based on its high bioethanol production efficiency from different types of residues [2,4,53], while S. *cerevisiae* was used as the most exploited yeast strain for sugar fermentation to bioethanol. In Figure 5 and Figure 6, the ethanol concentration from the pretreated WS during fermentation of 48 h with different mono-cultures and co-cultures, respectively, in SSF, is presented.

It is obvious that pretreatment approach D led to the highest ethanol concentrations using either mono-cultures or co-cultures, compared to the other pretreatment approaches. Regarding mono-cultures, *W. anomalus* led to the production of 6.4 ± 0.2 g ethanol/L and *S. cerevisiae* to the production of 5.4 ± 0.2 g/L, while the C5 strains, *P. stipitis* and *P. tannophilus*, led to the production of 4.5 ± 0.2 and 3.8 ± 0.1 g/L, respectively. In the case of co-cultures, the use of *P. stipitis* with *W. anomalus* led to the production of 6.9 ± 0.3 g/L, while its co-existence with *S. cerevisiae* resulted in 6.3 ± 0.2 g/L. In general, utilization of co-cultures for bioethanol fermentation is considered advantageous over mono-cultures due to the synergistic action of the metabolic pathways of the involved microorganisms [34]. Among them, co-culture of *P. stipitis* with *S. cerevisiae* is the most commonly used due to similar operational parameters in which both strains ferment glucose toward bioethanol [54]. For instance, Dhabhai et al. [55] observed higher ethanol production when using co-cultures of both yeast species for the fermentation of synthetic glucose and xylose media, while Ntaikou et al. [34] also supported this observation when using food wastes as substrate.

*P. tannophilus* exhibited the lowest ethanol concentration compared to the other yeast strains when fermenting the whole slurries of all pretreatment approaches. Similar results were obtained in previous studies, which compared *P. stipitis, S. cerevisiae* and *P. tannophilus* in fermentation experiments of the slurries after pretreatment of grass lawn with H_2_SO_4_ under SSF [8]. Contrary to these results, *P. tannophilus* was found to lead to improved bioethanol yields when compared to other yeast strains for the fermentation of hydrolysates resulting from biologically pretreated wheat straw [36].

In all cases, pretreatment approach B (sole H_2_O_2_) led to the production of the lowest ethanol concentration, either in mono- or co-cultures, compared to the other pretreatment approaches. This was anticipated based on the chemical (Figure 1b) and structural changes (Figure 3 and Figure 4) that treatment with H_2_O_2_ caused, which calls for the necessity of its combination with NaOH so as to increase the pH. Conversely, pretreatment with NaOH exhibited quite high ethanol concentrations, which was also justified by the partial hemicellulose and lignin removal that was observed (Figure 1b and Figure 3), thus making cellulose more accessible to enzymatic attack during SSF. 

**Figure 7:**
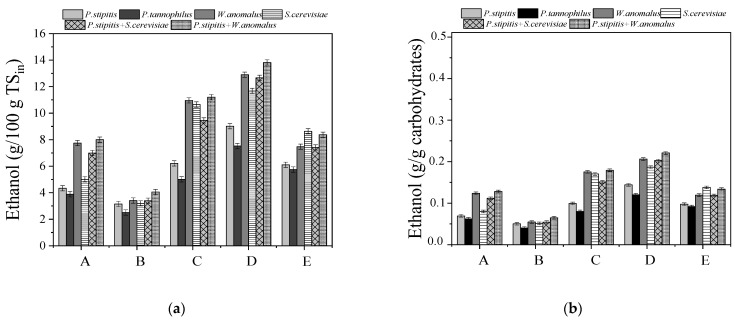
Ethanol yields expressed as (**a**) g/100 g TS_in_ (in: initial biomass) and (**b**) g/g, obtained after 48 h of fermentation of the whole pretreatment slurries, using *Pichia stipitis, Pachysolen tannophilus, Wickerhamomyces anomalus* X19, *Saccharomyces cerevisiae,* co-culture of *Pichia stipitis* with *Saccharomyces cerevisiae* and *Pichia stipitis* with *Wickerhamomyces anomalus* X19. Regarding pretreatments, (A) NaOH (0.5% *w*/*v*), (B) H_2_O_2_ (0.5% *v*/*v*), (C) mixture of NaOH (0.5% *w*/*v*) and H_2_O_2_ (0.5% *v*/*v*) at ratio 1:1, (D) initially NaOH (0.5% *w*/*v*) followed by H_2_O_2_ (0.5% *v*/*v*), and (E) initially H_2_O_2_ (0.5% *v*/*v*) followed by NaOH (0.5% *w*/*v*).

In Figure 7, the ethanol production yields, expressed as g/100 g TS_in_ (in: initial biomass) or per g of available carbohydrates (based on the initial content of cellulose and hemicellulose) contained in untreated WS, are presented. The ethanol yield could be compared to the theoretical maximum ethanol yield of 0.511 g/g (which is referred to the ethanol produced to total consumed carbohydrates), considering that all the available cellulose and hemicellulose were consumed. This could not had happened especially in the case of fermentations with the C5 strains, which do not have the ability to ferment xylose or arabinose, coming from hemicellulose degradation. 

Similar to the ethanol concentrations, the ethanol yields were higher in the case of pretreatment approach D, where WS was initially subjected to NaOH (0.5% *w*/*v*) for 24 h and then to H_2_O_2_ (0.5% *v*/*v*) for an extra 24 h. In addition, apart from *P. tannophilus* and *P. stipitis,* the other mono- or co-cultures exhibited ethanol yields in the range of 11.67 ± 0.21 to 13.81 ± 0.20 g/100 g TS (in all cases, statistical difference observed for fermentation with *S. cerevisiae, p* < 0.05). When comparing the ethanol yields, in the case of co-culture of *P. stipitis* with *W. anomalus*, the ANOVA results showed that there were statistical differences for pretreatments A, C, D and E. From the two-sample *t*-test statistical analysis, it was found that there were significant differences between pretreatments A vs. C, C vs. D, and A vs. D in ethanol production, while there was no significant difference for pretreatments A vs. E.

It should be emphasized that despite the existence of phenolic compounds in the pretreatment slurries, released due to lignin degradation, all pretreatment approaches led to high ethanol yields. Phenolic compounds at a specific concentration might have a toxic effect on yeasts and bacteria, causing cell disturbances [19]. However, the experimental results obtained showed that there was no toxic effect at the conditions tested, even in the case of pretreatment approach D, which was correlated with the highest phenolic concentration. The ethanol yields obtained in the present study are in line with the respective ones reported in other studies, using the whole hydrolyzed slurry after thermochemical pretreatment of lignocellulosics, in SSF. For instance, Antonopoulou [8] reported 8.83 ± 0.05, 8.82 ± 0.04 and 9.05 ± 0.42 g ethanol/100g TS, respectively, for the fermentation of the whole slurry obtained after acid pretreatment of grass lawn using *P. stipitis, P. tannophilus* and *S. cerevisiae.* In addition, ethanol yields of 4.54 ± 0.39 to 10.48 ± 0.04 g/100g TS were observed for the fermentation with *P. stipitis* of the slurry after acid and alkali pretreatments of sunflower straw [56]. However, exploiting other processing schemes, i.e., separate hydrolysis and fermentation (SHF) or separation of the slurry into two fractions (a liquid fraction rich in C5 sugars and a solid fraction containing cellulose) and fermentations into different reactors at the optimum conditions for each process, could be beneficial for the ethanol yields [4,8]. Conversely, from an engineering point of view, the use of the whole pretreatment slurry without separation and the fermentation/hydrolysis in the same reactor seems more attractive. A detailed techno-economical assessment is needed in order to select the best process scheme that will combine the optimum ethanol yields and the minimum processing cost. 

## 3. Materials and Methods 

### 3.1. Willow Sawdust

WS was collected during willow bark trimming in Greece, and the procedure of milling and sieving at a final size of 0.7 mm that followed is described in Alexandropoulou et al. [16]. WS powder was then air-dried for almost a week before its use in the experiments. 

### 3.2. Pretreatment Methods Tested 

Suspensions of air-dried WS at 5% solids loading (5 g TS at 100 mL chemical solution) were treated with (A) dilute solution of NaOH (0.5% *w*/*v*), (B) dilute solution of H_2_O_2_ (0.5% *v*/*v*), and (C) a mixture of NaOH (0.5% *w*/*v*) and H_2_O_2_ (0.5% *v*/*v*) in volumetric ratio of 1:1. All pretreatments were performed at 80 °C for 24 h, as in previous studies with alkaline pretreatments on lignocellulosics [8,56]. The WS was also subjected to a two-stage pretreatment approach: (D) initially treated with NaOH (0.5% *w*/*v*) at 80 °C for 24 h and then with H_2_O_2_ (0.5% *v*/*v*) at 80 °C for an extra 24 h, and (E) initially with H_2_O_2_ (0.5% *v*/*v*) at 80 °C for 24 h and then with NaOH (0.5% *w*/*v*) at 80 °C for another 24 h. All pretreatment experiments were performed in duplicate. After pretreatment, the whole slurry was neutralized with 6 N HCl and was used for the fermentation experiments.

### 3.3. Yeasts and Growth Conditions

The fermentation tests were performed using the yeasts *S. sereviceae*, CECT 1332, *P. stipitis*, CECT 1922 and the non-conventional yeast *W. anomalus X19 (P. anomala*, strain X19, MH237950.1) [2,53]. The growth media used and the procedure of the inoculation followed are described in detail in Ntaikou et al. [34,53]. Pre-cultures were prepared in Yeast Extract–Peptone–Dextrose (YPD) medium (20 g/L glucose, 10 g/L yeast extract, 10 g/L peptone, pH adjusted to 5.5 using 4 N HCl) and were incubated overnight at 30 °C, 150 rpm, until reaching OD_550_ ~2.000 (corresponding to 4 g/L). For the co-cultures of *S. cerevisiae* or *W. anomalus X19* with *P. stipitis*, equal volumes of the cultures were centrifuged, and the cells contained were harvested and used as an inoculum for the fermentation experiments. All inocula were used at 5% *v*/*v* of the final fermentation volume of each experiment, leading to an initial biomass concentration of 0.2–0.25 g/L.

### 3.4. Fermentation Tests

Fermentation tests were carried out using the whole slurries obtained from all pretreatment methods tested, via SSF, through the use of a commercial cellulase blend (CE) (Cellic CTec2-CEL, Sigma-Aldrich, Saint Louis, MO, USA) at 30 FPU/g TS and pH = 4.8, at 30 °C, as described in Ntaikou et al. [53]. The experiments were carried out in duplicate in 160 mL serum vials with a working volume of 25 mL and incubation at 150 rpm and 30 °C in batch mode. The vials were sealed with rubber stoppers and were equipped with 0.22 μm filters for CO_2_ venting. In all experiments, cells were harvested from pre-cultures by centrifugation at 6000 rpm for 10 min and were suspended in mineral solution containing KH_2_PO_4_, MgCl_2_·6H_2_O and (NH_4_)_2_SO_4_, each at concentrations of 1 g/L. The initial pH was set to 5.0 via NaOH or HCl solution (6 N).

### 3.5. Analytical Methods

TS and VS were measured according to standard methods [57]. The lignocellulosic content before and after pretreatments was quantified according to the National Renewable Energy Laboratory (NREL)’s standard laboratory analytical procedure (LAP) [58,59]. Soluble carbohydrates, reducing sugars, and phenolic compounds were quantified according to the methods of DuBois [60], Miller [61] and Folin and Ciocalteu [62], respectively. Ethanol concentration was measured in the HPLC-RI, as described in Antonopoulou et al. [56]. SEM images were obtained as described in Antonopoulou et al. [19], while FTIR spectral measurements were collected using an Alpha II spectrometer (Bruker, Billerica, MA, U.S) with a diamond ATR crystal. All spectra were recorded in the range of 4000–400 cm^−1^, with an average of 34 scans and a spectral resolution of 4 cm^−1^ in transmittance mode.

### 3.6. Calculations

The material recovery (MR) and material loss (ML) due to the pretreatments is given by Equations (1) and (2):

(1) $$\mathrm{ML}=\frac{{\mathrm{TS}}_{\mathrm{initial}}-{\mathrm{TS}}_{\mathrm{final}}\left(g\right)}{{\mathrm{TS}}_{\mathrm{initial}}\;\left(g\right)}\times 100$$

(2) MR=(1−ML)×100

where TS_initial_ and TS_final_ are the TS before and after pretreatments.

The reduction of lignin (where delignification efficiency can be calculated), cellulose and hemicellulose is given by Equation (3):

(3) $$\mathrm{Ci}\;\mathrm{loss}=\frac{{\mathrm{Ci}}_{\mathrm{initial}\;}-{\;\mathrm{Ci}}_{\mathrm{final}}}{{\mathrm{Ci}}_{\mathrm{initial}}}$$

where Ci_initial_ and Ci_final_ are the initial and final concentrations of lignin, cellulose and hemicellulose before and after pretreatments, respectively. Ci_final_ is expressed in terms of ${\mathrm{TS}}_{\mathrm{initial}}$, taken into account the MR during pretreatment. Thus, Ci_final_ is calculated based on Equation (4), where gi are the g of component i:

(4) $$\mathrm{Ci}\;\mathrm{final}={\mathrm{Ci}}_{\mathrm{final}\;}\left(\frac{g\;i}{100\;\mathrm{gTS}\;\mathrm{pretreated}\;\mathrm{biomass}}\right)\times \;\mathrm{MR}\;\left(\frac{g\;\mathrm{TS}\;\mathrm{pretreated}\;\mathrm{biomass}}{g\;\mathrm{TS}\;\mathrm{initial}\;\mathrm{biomass}}\right)$$

The saccharification degree due to the pretreatments was estimated using Equation (5):

(5) $$\mathrm{Saccharification}\;\mathrm{degree}=\frac{\mathrm{Reducing}\;\mathrm{sugars}\;\left(g/100g\;\mathrm{TS}\right)}{\mathrm{Soluble}\;\mathrm{carbohydrates}\;\left(g/100g\;\mathrm{TS}\right)}$$

using the concentration of soluble carbohydrates and reducing sugars released due to pretreatment. 

The saccharification efficiency due to solubilization of hollocellulose (cellulose and hemicellulose) contained in WS due to different pretreatment methods was estimated using Equation (6):

(6) $$\mathrm{Saccharification}\;\mathrm{efficiency}=\frac{\mathrm{Soluble}\;\mathrm{sugars}\;\left(g/100g\mathrm{TS}\right)}{\mathrm{Initial}\;\mathrm{hollocellulose}\;\mathrm{of}\;\mathrm{WS}\;\left(g/100g\mathrm{TS}\right)}\;$$

where the amount of soluble sugars is estimated as the concentration of soluble carbohydrates in the pretreated WS slurries (g/100 g TS), and the initial holocellulose of WS is the sum of cellulose and hemicellulose before pretreatment (g/100 g TS).

Meanwhile, the ethanol yield in terms of g/g carbohydrates was estimated via Equation (7)

(7) $$\mathrm{Ethanol}\;\mathrm{yield}=\frac{\mathrm{Ethanol}\;\left(g/100g\;\mathrm{TS}\right)}{\mathrm{Initial}\;\mathrm{hollocellulose}\;\mathrm{of}\;\mathrm{WS}\;\left(g/100g\;\mathrm{TS}\right)}\;$$

### 3.7. Statistical Analysis 

Two-sample *t*-test and one-way analysis of variance (ANOVA) with a threshold *p*-value of 0.05 was applied to analyze statistical differences using excel software. 

## 4. Conclusions

The experimental results showed that when willow sawdust (WS) was subjected to a two-stage pretreatment approach of NaOH (0.5% *w*/*v*)/H_2_O_2_ (0.5% *v*/*v*), high delignification (38.3 ± 0.1%) and saccharification efficiency (31.7 ± 0.3%) were observed, accompanied by high ethanol concentrations and yields, either using mono-cultures or co-cultures of ethanol-producing yeast strains. On the contrary, pretreatment with H_2_O_2_ (0.5% *v*/*v*) alone led to the lowest ethanol yields due to the minor impact of this treatment on the main chemical and structural WS characteristics. Regarding the yeast strains used, *Saccharomyces cerevisiae*, *Wickerhamomyces anomalus X19* and co-cultures of *P. stipitis* with either *S. cerevisiae* or W*. anomalus X19* exhibited high ethanol yields (11.67 ± 0.21 to 13.81 ± 0.20 g/100 g total solids (TS)), after the two-stage NaOH/H_2_O_2_ pretreatment, indicating the effectiveness of this treatment for ethanol production from WS. In order to decide which is the best pretreatment approach, a detailed techno-economical assessment is needed that will take into account the ethanol yields and the minimum processing cost.

## Figures and Tables

**Table 1:** The main characteristics of WS used in the study.

Composition	Value (g/100 g TS)
Total Solids (TS) ^a^	93.4 ± 0.2
Volatile Solids (VS)	94.1 ± 0.2
Cellulose	33.4 ± 1.1
Hemicellulose	21.5 ± 0.9
Lignin	29.1 ± 0.6
Extractives	3.0 ± 0.2
Ash	5.9 ± 0.2

^a^ is expressed as g TS/100 g wet weight.

**Table 2:** Saccharification degree expressed as the ratio of reducing sugars to soluble carbohydrates and saccharification efficiency.

Pretreatment—Symbol	Reducing/Soluble (% g/g)	Saccharification Efficiency (% g/g)
NaOH–A	28.3 ± 0.2	24.0 ± 0.2
H_2_O_2_–B	22.2 ± 0.1	13.8 ± 0.1
Mixture NaOH/H_2_O_2_–C	30.9 ± 0.5	26.1 ± 0.2
NaOH/H_2_O_2_–D	29.5 ± 0.0	31.7 ± 0.3
H_2_O_2_/NaOH–E	30.5 ± 0.5	27.6 ± 0.2

**Table 3:** Characteristic IR bands of samples studied in the fingerprint region.

Wavenumber (cm^−1^)	Functional Group
1732	C=O stretching (hemicellulose)
1598	C=C stretching of the aromatic ring (lignin)
1510	C=C stretching of the aromatic ring (lignin)
1230	Syringyl ring and C–O stretch in lignin
898	Asymmetrical out of phase ring stretching (crystalline cellulose)

## Data Availability

The datasets discussed and analyzed in the current study are available from the corresponding author upon request.

## Sample Availability

Samples of the compounds are available from the authors.
